# The Cao-Xiang-Wei-Kang formula attenuates the progression of experimental colitis by restoring the homeostasis of the microbiome and suppressing inflammation

**DOI:** 10.3389/fphar.2022.946065

**Published:** 2022-09-20

**Authors:** Wei Yu, Qi Li, Changlei Shao, Yijia Zhang, Cai Kang, Yang Zheng, Xihao Liu, Xincheng Liu, Jing Yan

**Affiliations:** Department of Physiology, Jining Medical University, Jining, Shandong, China

**Keywords:** ulcerative colitis, inflammatory bowel disease, dysbiosis, mucosal healing, Cao-Wei-Kang formula

## Abstract

Inflammatory bowel disease (IBD) is pathologically characterized by an immune response accommodative insufficiency and dysbiosis accompanied by persistent epithelial barrier dysfunction. The Cao-Xiang-Wei-Kang (CW) formula has been utilized to treat gastrointestinal disorders in the clinic. The present study was designed to delineate the pharmacological mechanisms of this formula from different aspects of the etiology of ulcerative colitis (UC), a major subtype of IBD. Dextran sodium sulfate (DSS) was given to mice for a week at a concentration of 2%, and the CW solution was administered for 3 weeks. 16S rRNA gene sequencing and untargeted metabolomics were conducted to examine the changes in the microbiome profile, and biochemical experiments were performed to confirm the therapeutic functions predicted by system pharmacology analysis. The CW treatment hampered DSS-induced experimental colitis progression, and the targets were enriched in inflammation, infection, and tumorigenesis, which was corroborated by suppressed caspase 3 (Casp3) and interleukin-1b (IL-1b) and increased cleaved caspase 3 expression and casp-3 activity in the colon samples from colitis mice subjected to the CW therapy. Moreover, the CW therapy rescued the decreased richness and diversity, suppressed the potentially pathogenic phenotype of the gut microorganisms, and reversed the altered linoleic acid metabolism and cytochrome P450 activity in murine colitis models. In our *in vitro* experiments, the CW administration increased the alternative activation of macrophages (Mφs) and inhibited the tumor necrosis factor-α (TNFα)-induced reactive oxygen species (ROS) level and subsequent death in intestinal organoids (IOs). We propose that the CW formula alleviates the progression of murine colitis by suppressing inflammation, promoting mucosal healing, and re-establishing a microbiome profile that favors re-epithelization.

## Introduction

Inflammatory bowel diseases (IBDs) are systemic diseases and not only impair the gut functions but also influence extraintestinal organs, such as the liver, kidney, and eyes (Rogler et al., 2021). The pathophysiological factors include genetic susceptibility, environmental pollution, pathogen invasion, and dietary habits. Ulcerative colitis (UC) is a major subtype of IBD and predominantly occurs in the colon, accompanied by bloody stool, weight loss, abdominal pain, and a higher risk of developing colitis-associated carcinogenesis (CAC). To successfully intervene with the progression of UC, therapy should take all these pathological symptoms into account. In other words, resolution of inflammation and inflammation-compromised integrity of the colon epithelium, as well as recovery of healthy gut flora and rescued intestinal stem cell viability, are pivotal for UC treatment. Therefore, multi-target drugs exhibit stronger clinical efficacy than exquisitely selective compounds. Derived from the oldest Chinese medical book “Shang Han Lun” and evolving with the modern medical technique, traditional Chinese medicine (TCM) has a long historical application in clinics and is a golden resource for developing multi-target drugs. However, the pharmacological mechanisms of many efficacious drugs need extensive experimental evidence to corroborate the efficacy and guarantee of low toxicity. The emergence of system pharmacology and omics technology helps resolve the difficulty in explaining the synergistic and counteracting effects of multiple herbs included in each TCM formula, providing a chance for the development of TCM.

The Cao-Xiang-Wei-Kang (CW) formula included in Chinese Pharmacopoeia 2020 is composed of *Senna tora* (L.) Roxb (SR), oyster shell (OS), *Gallus gallus domesticus Brisson* (GB), cuttlebone (Cb), *Ferula sinkiangensis* K.M.Shen (FS), and *Dolomiaea berardioidea* (Franch.) C. Shih (DC) (Table 1). This investigation was designed to determine the efficacy of the CW formula from combination logic to experimental evidence by integrating network pharmacology with omics technology and *in vitro* and *in vivo* experiments.

**TABLE 1:** The ingredients of the formula.

Scientific Name of the herb	Chinese name	Weight %
Senna tora (L.) Roxb	Jue Ming Zi	35%
oyster shell	Mu Li	20%
Gallus gallus domesticus Brisson	Ji Nei Jin	15%
Cuttlebone	Hai Piao Xiao	15%
*Ferula sinkiangensis K.M.Shen*	A Wei	10
*Dolomiaea berardioidea (Franch.) C.Shih*	Mu Xiang	5

## Materials and methods

### Network pharmacology

A CW–component–target network was constructed by active components with a cut-off of drug-likeness (0.18) and oral bioavailability (20%). All the plant names have been checked with http://www.theplantlist.org. Based on the database of Laboratory of Systems Pharmacology (Ru et al., 2014), the active components of SR and DC were collected. The active components of OS, Cb, GB, and FS were acquired, according to the published articles (Lee et al., 2010; Pan et al., 2016; Scandiffio et al., 2018; Guo et al., 2020; Kintsu et al., 2020). According to these active components, the targets were obtained from PubChem, and the genes of ulcerative colitis (UC) were collected from GeneCards (Stelzer et al., 2016), Online Mendelian Inheritance in Man (OMIM) (Amberger and Hamosh, 2017), DrugBank (Wishart et al., 2018a), PharmGkb (Barbarino et al., 2018), and Statistics of Therapeutic Target Database (TTD). Based on the commonly shared genes, Gene Ontology (GO) and Kyoto Encyclopedia of Genes and Genomes (KEGG) Pathway Enrichment analyses were conducted (The Gene Ontology Consortium, 2019). The herb–ingredient–target (HIT) interaction network and a protein–protein interaction (PPI) network with a confidence score ≥ 0.7 based on the Search Tool for Retrieval of Interacting Genes/Proteins database were computed by CytoNCA (Tang et al., 2015), which calculates the median values of betweenness centrality (BC), closeness centrality (CC), degree centrality (DC), local average connectivity (LAC), eigenvector centrality (EC), and network centrality (NC).

### Cao-Xiang-Wei-Kang formula preparation

The CW formula was prepared by mixing the powder of SR, OS, GB, Cb, FS, and DC at a ratio of 7:4:3:3:2:1, respectively. Briefly, the FS powder was diluted in water and mixed with other herbs. The dried drug was smashed and given to mice or subjected to mass spectrometry (MS) detection. To validate the components in the formula, a connected system of LC-30 (Shimadzu)-Hybrid Quadrupole Time-of-Flight MS (TOF MS) with an electrospray ionization source (ESI) was used. The mobile phase system had solution A (acetonitrile) and equate B (0.1% HCOOH-H_2_O): 25 min (A:20%: B:80%), 50 min (A:35%: B:65%), 9 min (A:90%: B:10%), and 7 min (A:20%: B:90%). Data were processed in information-dependent acquisition (IDA) with a high-sensitivity mode.

### Experimental colitis murine models with the administration of Cao-Xiang-Wei-Kang

Experiments were approved by the guidelines of the Institutional Animal Care and Use Committee of Jining Medical University in China (SYXK-Shandong province-2018-0002).

Mice (Pengyue Animal Center of Shandong province, China) (male, about 22 g, 2 months old) were given 2% dextran sodium sulfate (DSS, MP Biomedicals) for 1 week, and then, the CW formula was administered for 21 days. The dose of the human being was 120 mg/kg per day based on the Chinese Pharmacopoeia 2020 version, and correspondingly, murine was 1.44 g/kg calculated by the Meeh–Rubner formula: $Skin\;surface\;area=Mass\;Coefficients\times \left(\frac{Weight\times \frac{2}{3}}{10000}\right)$ . An amount of 36 mg (equal to 1.44 g/kg) (the high dose) or 18 mg (equal to 0.7 g/kg) (the low dose) CW diluted in 200 μl water was administered each day. The positive control of the study was mesalazine (MedChemExpress, China) (Veloso et al., 2021). Every 15 mice were grouped, and five groups were set as follows: 0.9% saline, 2% DSS, the low-CW-DSS group, high-CW-DSS group, and mesalazine group (200 mg/kg). Isoflurane (RWD Life Science, Shenzhen City, China) was used for gas anesthetization. The histopathological score was determined: weight loss (normal: 0; <5%: 1; 5%–10%: 2; 10%–20%: 3; >20%: 4); feces (normal: 0; soft: 1; very soft: 2; liquid: 3); blood test (no blood within 2 min: 0; positive in 10 s: 1; light purple within 10 s: 2; heavy purple within 10 s: 3) (Leagene, China); and histological indices (destruction of the epithelium, immune cell infiltrates, edema, and crypt loss, each for 1).

The colon samples were soaked in paraformaldehyde (PFA) for 72 h, embedded in paraffin and then cut into 3-μm thick slices, and subjected to hematoxylin and eosin (H&E) staining.

### Serum isolation

After the high-dose treatment of CW, we collected the serum from colitis mice in 2 hours for the following experiments. To examine the toxicity, NCM460 cells, an epithelial cell line derived from the normal colon of a 68-year-old Hispanic male, were treated with serum and subjected to an MTT assay (3- [4,5-dimethylthiazol-2-yl]-2,5-diphenyl tetrazolium bromide) and cellular immunofluorescence assay with PI staining (Abcam, United States).

### Enzyme-linked immunosorbent assay

Enzyme-linked immunosorbent assay (ELISA) kits were bought from Abcam and were utilized to examine the cytokine concentrations in the serum: TNFα (sensitivity: 0.1 pg/ml; range: 47–3,000 pg/ml) and IL-1b ELISA kits (sensitivity: 15.2 pg/ml; range: 28.1–1,800 pg/ml).

### 16S rRNA gene amplicon sequencing data analysis

The feces and mucus within the colon were collected and mixed and processed by 16S rRNA gene sequencing. The raw data were filtered by Trimmomatic (Bolger et al., 2014), and paired-end reads were produced after chimera removal by UCHIME. Corrected paired-end reads generated circular consensus sequencing (CCS) reads, which were distinguished based on the barcode sequences. After removing chimeras, an OTU (operational taxonomic unit) analysis was performed with a similarity >97%. Species annotation and taxonomic analysis, diversity analysis including alpha and beta diversity, significant difference analysis, and functional prediction were performed. Differential analysis among groups was calculated by the *t*-test. The rarefaction curve and Shannon curve were plotted using Mothur and R packages. The rank abundance curve (Guo et al., 2020) was generated by ranking the features in each sample based on abundance and plotting them in abundance rank (X-axis) and relative abundance (Y-axis) (Salari et al., 2016). Beta diversity analysis was processed by QIIME software to compare species diversity between different samples utilizing binary jaccard, which was an OTU-based algorithm. Principal coordinates analysis (PCoA) was drawn by the R language tool, and PERMANOVA (Adonis) created by the vegan package was used to analyze whether there was a significant difference in beta diversity between samples from different groups (Xie et al., 2020). LEfSe [Linear Discriminant analysis (LDA) Effect Size] was conducted to identify biomarkers with statistical difference between different groups (Zhou et al., 2020). Metastatsconducted a *t*-test on species richness data between groups at the taxonomic level of the genus (Hu et al., 2018). Krona, as a powerful metagenomic visualization tool, demonstrates species annotation. Each fan means the corresponding annotation’s proportion (Ondov et al., 2011). PICRUSt (Phylogenetic Investigation of Communities by Reconstruction of Unobserved States) analysis was conducted to predict the alterations in KEGG pathways. The raw data were available with the accession number in SRA (PRJNA827781).

### Untargeted metabolomics

Fecal and mucus samples were treated with methanol and standard internal substances. After the ultrasound and frozen treatment, the samples were centrifuged. The supernatant was subjected to ultra-high-performance liquid chromatography (UHPLC) coupled with TOF-MS (Garcia and Barbas, 2011). Acquisition software (Analyst TF 1.7, AB Sciex) was utilized to assess the full scan survey MS data, which were then converted by ProteoWizard and generated the retention time (RT), mass-to-charge ratio (m/z) values, and peak intensity. According to the in-house MS2 database, substances were identified and determined by orthogonal projections to latent structures-discriminant analysis (OPLS-DA). The cut-off was *p* value < 0.05, fold change (FC) > 1, and Variable Importance in the Projection (VIP) > 1. The HMDB (Human Metabolome Database) (Wishart et al., 2018b) and KEGG database were utilized to annotate metabolites.

### Peritoneal macrophage isolation

Mice were anesthetized, and sterilized PBS was injected into the abdominal cavity. After massage, the liquids were isolated and centrifuged. The pellet was diluted in RPMI-1640 complete medium.

### Microparticle phagocytic experiment

Diluted microparticles (Thermo Fisher, United States) [fluorescein isothiocyanate (FITC) wavelength-488 nm] were incubated with peritoneal Mφs collected from anesthetized mice for 2 h at 37°C, were centrifuged and washed, and subjected to flow cytometry analysis.

### Wound healing assay

A density of 10^5^ NCM460 cells was seeded in the 24-well cell culture plate at a 37°C 5% CO_2_ incubator. When reaching 100% confluency, a scratch was made, and a transwell insert (Corning, 0.3 μm diameter, United States) carrying 10^4^ peritoneal Mφs was placed. After 24 h, the cells in five random fields were examined.

### Western blotting

Total protein (40 µg) was subjected to SDS-PAGE (sodium dodecyl sulfate–polyacrylamide gel electrophoresis) and transferred. After blockage, antibodies [CASP3, vascular endothelial growth factor A (VEGFa), IL-1b, IL-10, and arginase 1 (ARG1)] were incubated with the membrane for 24 h. After washing with PBS, secondary antibodies conjugated with horseradish peroxidase (HRP) were utilized, and an enhanced chemiluminescence (ECL) substrate was added to visualize the protein bands. All chemicals were bought from Invitrogen, United States.

### Caspase-3 activity assay

The colon samples were subjected to the Caspase-3 activity assay kit (Abcam, United States), and the absorbance was determined at an optical density at 405 nm (BioTek Instruments, Inc.).

### Intestinal organoid culture

The small intestine was cut into small pieces and washed with cold PBS until the solution became clear. After 35 min of digestion by the digestive solution (Yu et al., 2020), the filtered medium was centrifuged and cultured in a growth medium. After 9 days, tumor necrosis factor-α (TNFα) was added with or without the CW serum. After 24 h, IOs were incubated at 37°C with propidium iodide (PI)/Hoechst33342 (480 nm/630 nm) for 20 min. To measure mitochondrial stress, MitoSOX™ Red mitochondrial superoxide indicator (590 nm) was added. After 10 min at 37°C, cells were examined using a fluorescent microscope. Two dyes and culturing reagents were bought from Thermo Fisher and STEMCELL Technologies, respectively.

### Statistical analysis

Data are means ± SEM. *p < 0.05, **p < 0.01, and ***p < 0.001. Every experiment was repeated at least five times. Comparisons between two groups were analyzed by unpaired Student’s *t*-test. GraphPad 8.0 and R software were utilized.

## Results

### Therapeutic role of the Cao-Xiang-Wei-Kang formula against experimental colitis

**FIGURE 1:**
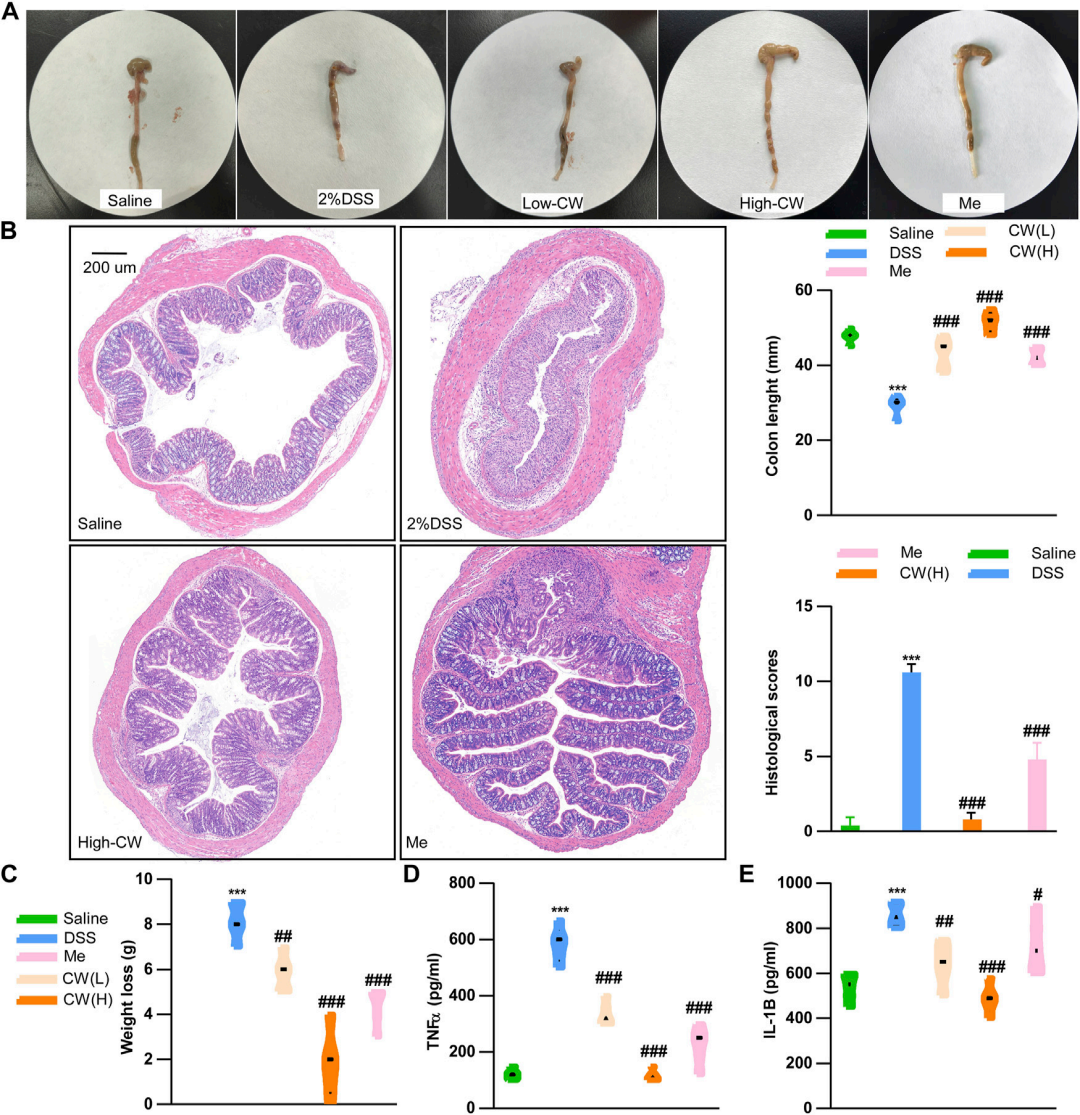
CW formula alleviates the progression of experimental colitis. Colon tissue and colon length **(A)**, HE staining and histological scores of the colon **(B)**, weight loss **(C)**, serum TNFα **(D),** and IL-1b **(E)** levels in ulcerative colitis (UC) murine models after the CW treatment (*n* = 15). *indicates a statistically significant difference from the saline group, and ^#^indicates a statistically significant difference from the DSS group.

We determined the chemical profile of the CW formula by UPLC-MS/MS and identified the presence of the ingredients (Supplementary Figure S1, Supplementary Table S1). DSS exacerbated weight loss, caused rectal bleeding, shortened the colon length, and induced edema. These symptoms were markedly alleviated by the CW therapy at a low and high dose (1.44 g/kg). As compared with the mesalazine treatment, the high dose of CW produced a longer colon (Figure 1A). H&E staining showed that the high dose of CW successfully rescued colitis-caused crypt loss and alleviated epithelial breach, as well as immune cell infiltrates, showing its superiority in mucosal healing and resolution of inflammation compared with mesalazine (Figure 1B). Of note, both doses of CW rescued weight loss (Figure 1C) and suppressed the DSS-evoked IL-1b and TNFα levels (Figures 1D,E).

### Herb–Ingredient–Target network of Cao-Xiang-Wei-Kang

**FIGURE 2:**
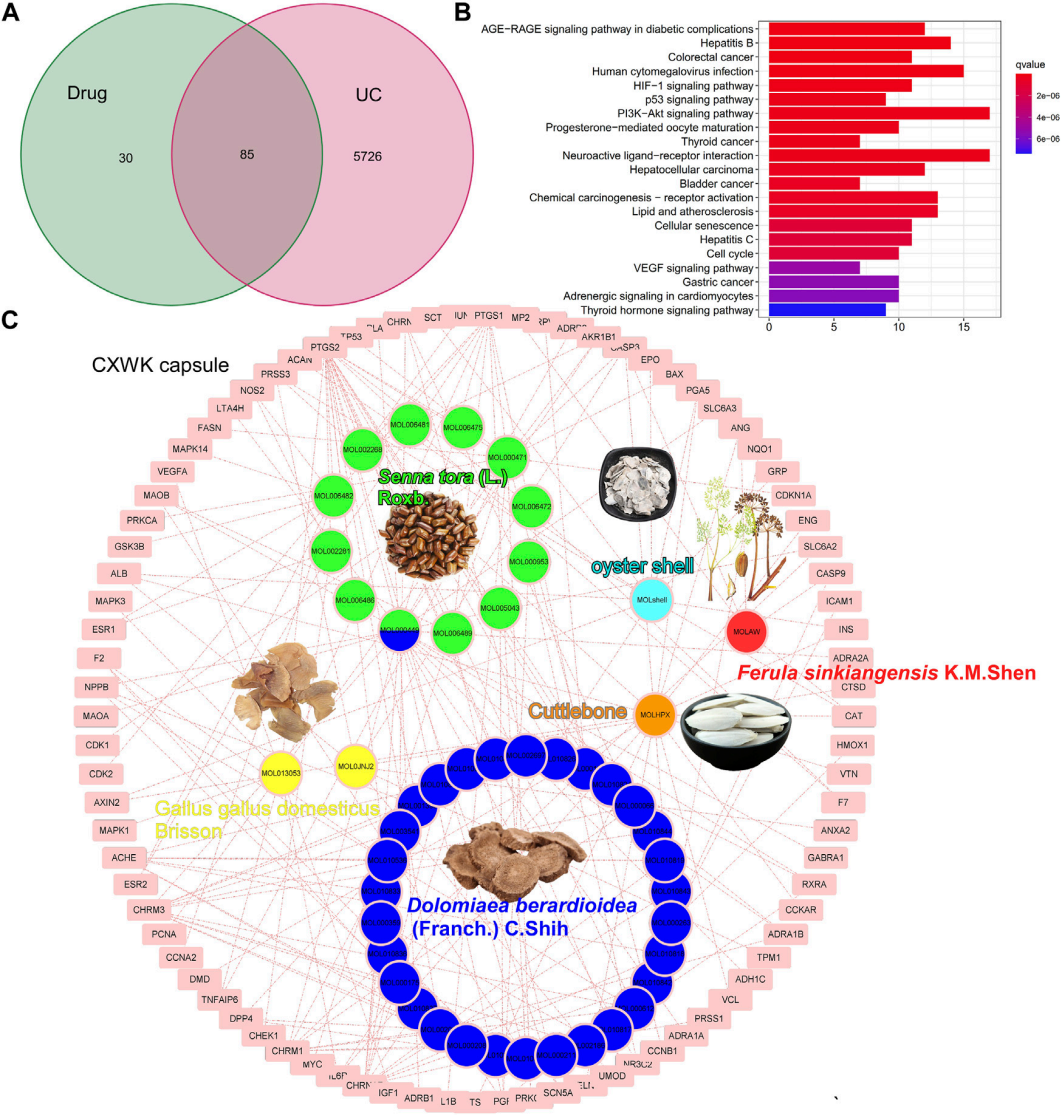
Component–target network of CW. Venn plot illustrates the shared targets between CW and UC **(A)**; KEGG analysis of the shared genes **(B)**; network of compounds and the targets in UC **(C)**.

The CW formula encompassed six herbs (Supplementary Table S2) and shared 85 putative targets with the acquired 5811 colitis-relevant genes (Figure 2A). A HIT network representing the interactions between active components and targeted genes was established: pink rectangle nodes represented colitis-associated genes, and nodes with different colors inside were active components (Figure 2C). Based on the database and published articles, 12 active components of SR, 30 of DC, and 2 of GB were identified, while OS, FS, and Cb had three components. Among the 115 targets of these components, 85 genes were associated with the progression of colitis.

**FIGURE 3:**
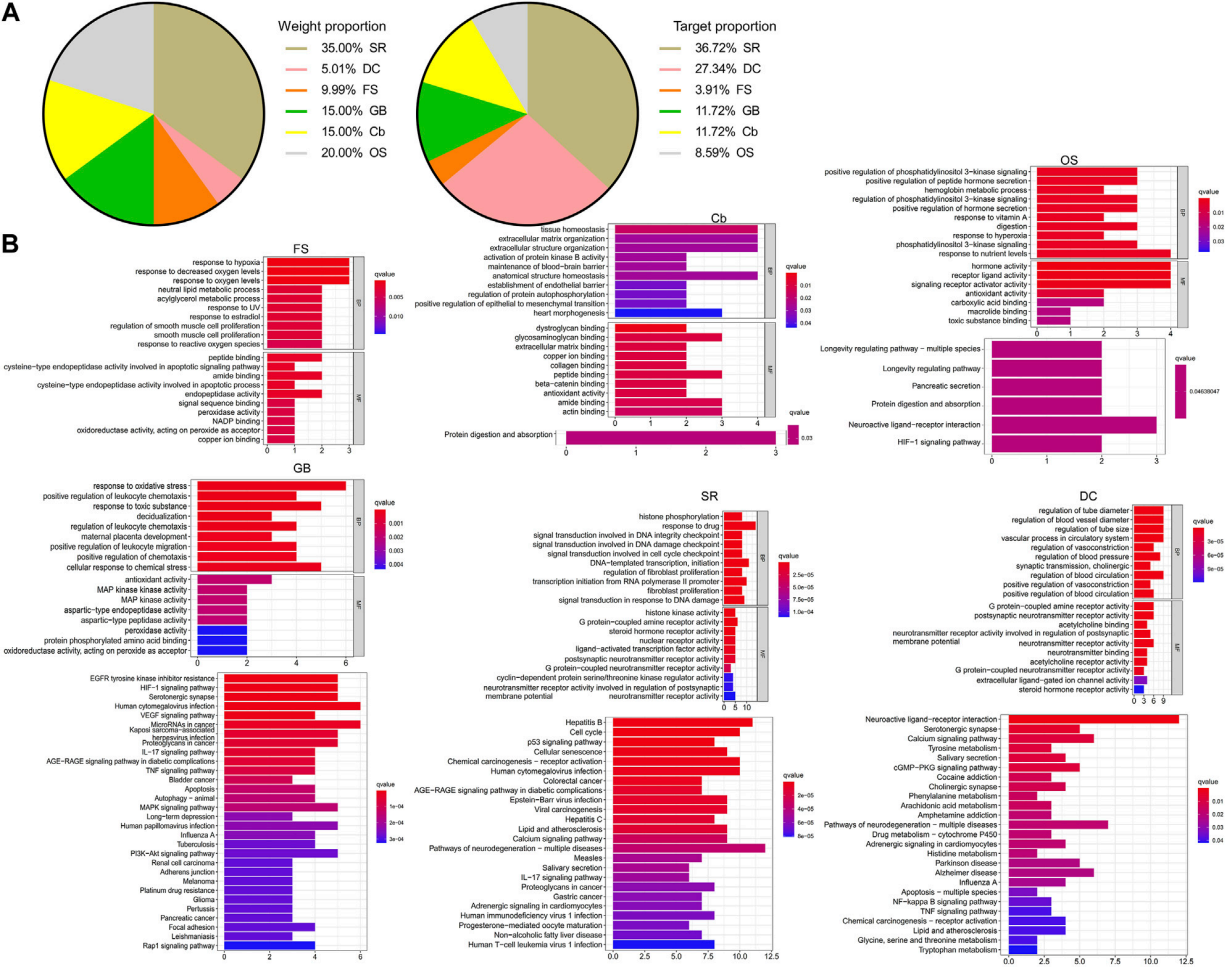
Pharmacological mechanisms of CW. Pie chart demonstrates the percentage of the target genes and weight percentage of CW ingredients **(A)**; GO and KEGG analysis show the predicted function of CW components **(B)**.

The KEGG analysis demonstrated that these shared genes were enriched in carcinogenesis, infections, neuroactive ligand–receptor interaction, and VEGF and HIF-1 signaling pathways (Figure 2B). As illustrated in the pie charts, and weight or target proportion, SR occupied much of the formula (Figure 3A). All the ingredients are associated with infection and CRC, as well as inflammatory responses (Figure 3B).

**FIGURE 4:**
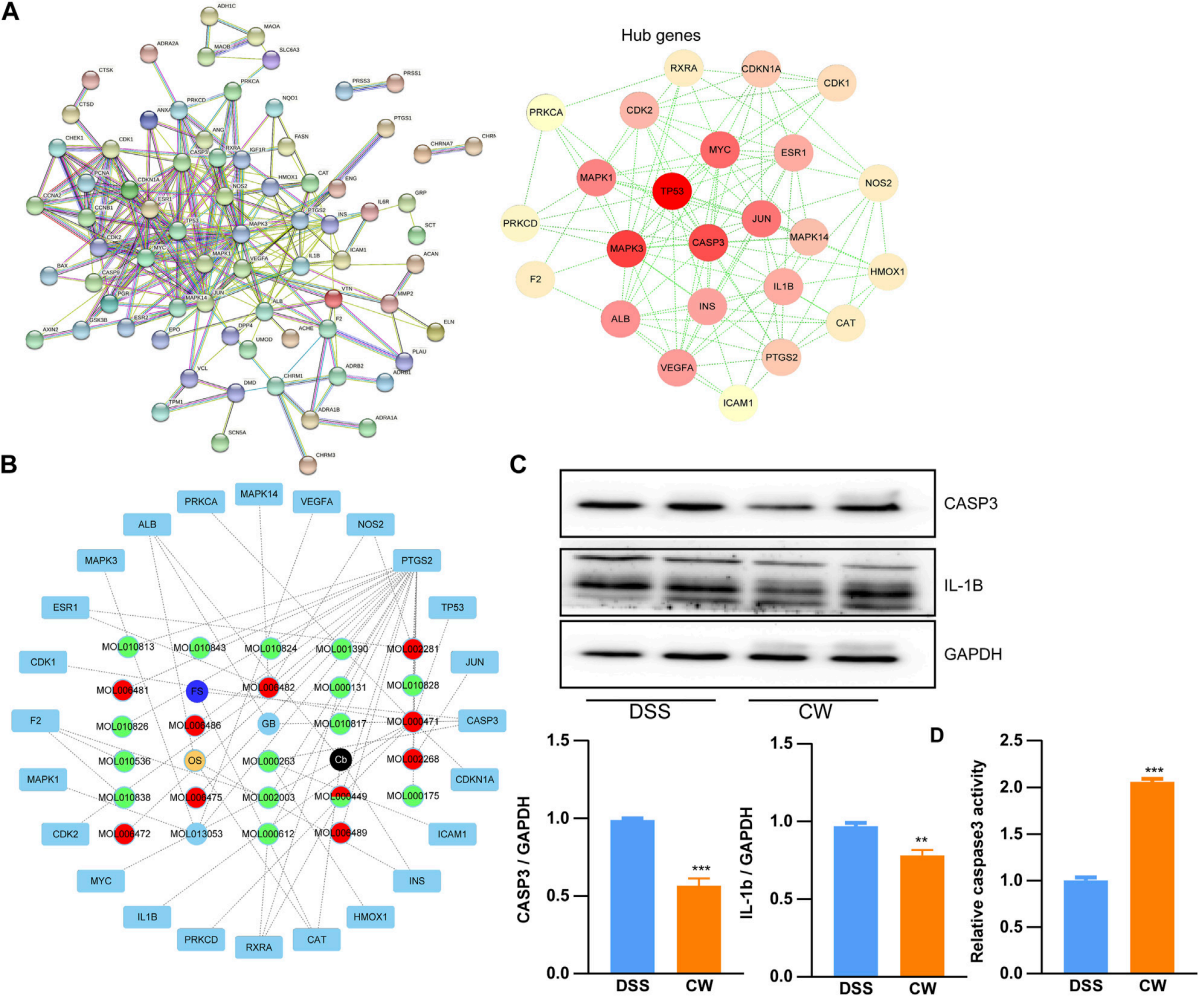
Protein–protein interaction network of the CW formula. Interaction network **(A)**; hub gene–component network **(B)**; and the expression of Casp3 and IL-1b, as well as the Casp3 activity in the colon samples from colitis mice with or without CW treatment **(C,D)** (*n* = 5). *indicates a statistically significant difference from the DSS group.

Based on the shared genes between colitis and the CW formula, a PPI network with a PPI enrichment *p*-value (<1.0e-16) was established utilizing the STRING database, and a sub-network composed of 24 genes is shown in Figure 4A with the median values (BC: 16.157288675, CC: 0.151452282, DC: 5, EC: 0.0531741455, LAC: 1.625, and NC: 2.5833333335). To predict the major active components in the formula, the calculated 24 hub genes and corresponding components were utilized to construct a component–hub gene network (Figure 4B). Western blotting confirmed that the high dose of CW treatment significantly suppressed the protein abundance of Casp3 and IL-1b, and increased the Casp3 activity (Figures 4C,D), indicating an alleviated inflammation by the CW formula.

### The Cao-Xiang-Wei-Kang formula rescued dysbiosis in murine colitis models

**FIGURE 5:**
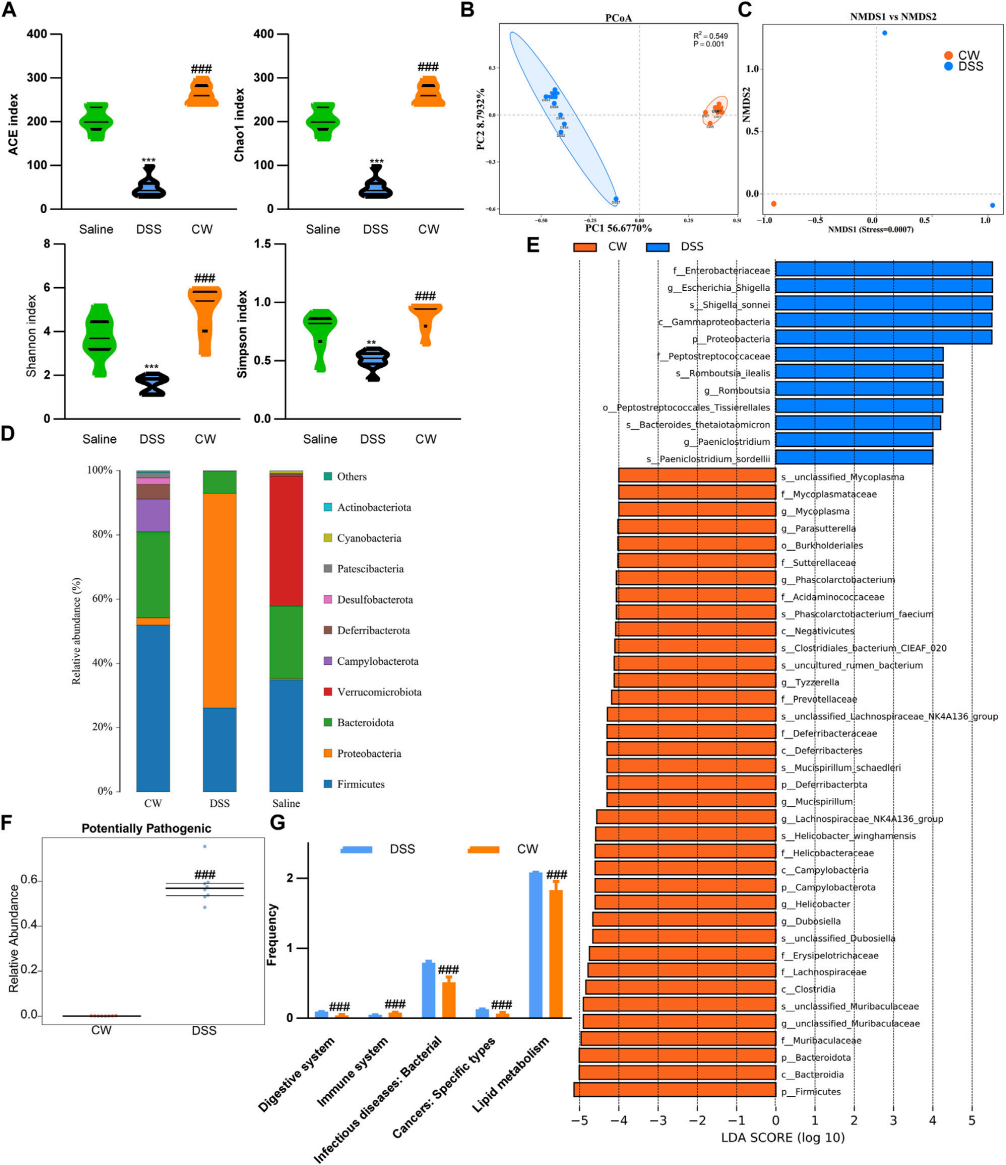
Alteration in the microbiota profile in UC murine models after CW treatment. Alpha-diversity of microbial communities in mice undergoing colitis and treated with CW **(A)**. PCoA **(B)** and NMDS **(C)** show the clustering of gut flora; microbiota composition at the phylum **(D)**; LEFSe **(E)** illustrates the abundance of bacterial species in experimental colitis murine models after CW administration; BugBase **(F)** and PICRUSt **(G)** prediction. *indicates a statistical difference from the saline group, and ^#^indicates a difference from the DSS group.

To determine the effect of the CW formula on microbiota composition, 16S rRNA gene sequencing was conducted utilizing the feces and mucus. All sample libraries covered >99%, suggesting a sufficient library size to represent most microbes (Supplementary Table S3). DSS treatment led to a marked decrease in the alpha-diversity of microbiota including Chao and Ace, and Shannon indexes, as well as the Simpson index, all of which were reversed by the CW therapy (1.44 g/kg) (Figure 5A). β-diversity was calculated by the binary Jaccard method. Principal Co-ordinates Analysis (PCoA) combined with PERMANOVA showed that CW treatment significantly altered the β-diversity of commensal microorganisms (Figure 5B). Non-metric multidimensional scaling analysis (NMDS) indicated the CW-DSS group clustered distinctly from the DSS group with a stress value of 0.02 (Figure 5C). At the phylum level, we observed an apparent reduction in Bacteroidota and Firmicutes in the DSS group compared with other groups, with an increase in Proteobacteria (Figure 5D). LEFSe showed that the CW treatment increased the relative abundance of the phyla Bacteroidota and Firmicutes, whereas decreased the prevalence of Proteobacteria at the phylum level. The administration of CW suppressed the abundance of species *Romboutsia ilealis*, *Bacteroides thetaiotaomicron*, *Paeniclostridium sordellii*, and *Escherichia*, and *Shigella*, while increased *Lachnospiraceae NK4A136*, *Phascolarctobacterium faecium, Mucispirillum schaedleri,* and *Helicobacter winghamensis* (Figure 5E). BugBase feature prediction calculated by Mann–Whitney–Wilcoxon analysis showed that the CW treatment significantly reversed the DSS-induced abundance of potentially pathogenic phenotype microorganisms (Figure 5F). PICRUSt analysis demonstrated that the administration of XQ inhibited infectious diseases and cancers, and lipid metabolism, controlled the digestive system, and improved the immune system (Figure 5G).

To examine the alterations in the metabolic profile, untargeted metabolomics was performed. OPLS-DAOPLA-DA showed that the metabolites between the saline and the DSS group were clustered distinctly between the two groups with Q2Y (0.894, 0.782) and R2Y (0.953, 0.953) (Supplementary Figure S2A). A Q2 value of more than 0.5 indicates the precise predictivity of the model. A permutation test was performed to validate the model, and R2 and Q2 should be higher than the permutated models with a vertical axis intersection of Q2 that was lower than zero. As illustrated, the intersection of Q2 was (0.0, −1.35) and (0.0,−1.35), which confirmed the validity of the OPLS-DA model (Supplementary Figure S2B). According to VIP, the top five regulated outlier metabolites in the DSS group are shown in (Supplementary Figure S2C, Supplementary Table S4). The KEGG analysis showed that DSS suppressed linoleic acid metabolism, whereas it increased the cytochrome P450 activity (Supplementary Figure S2D).

**FIGURE 6:**
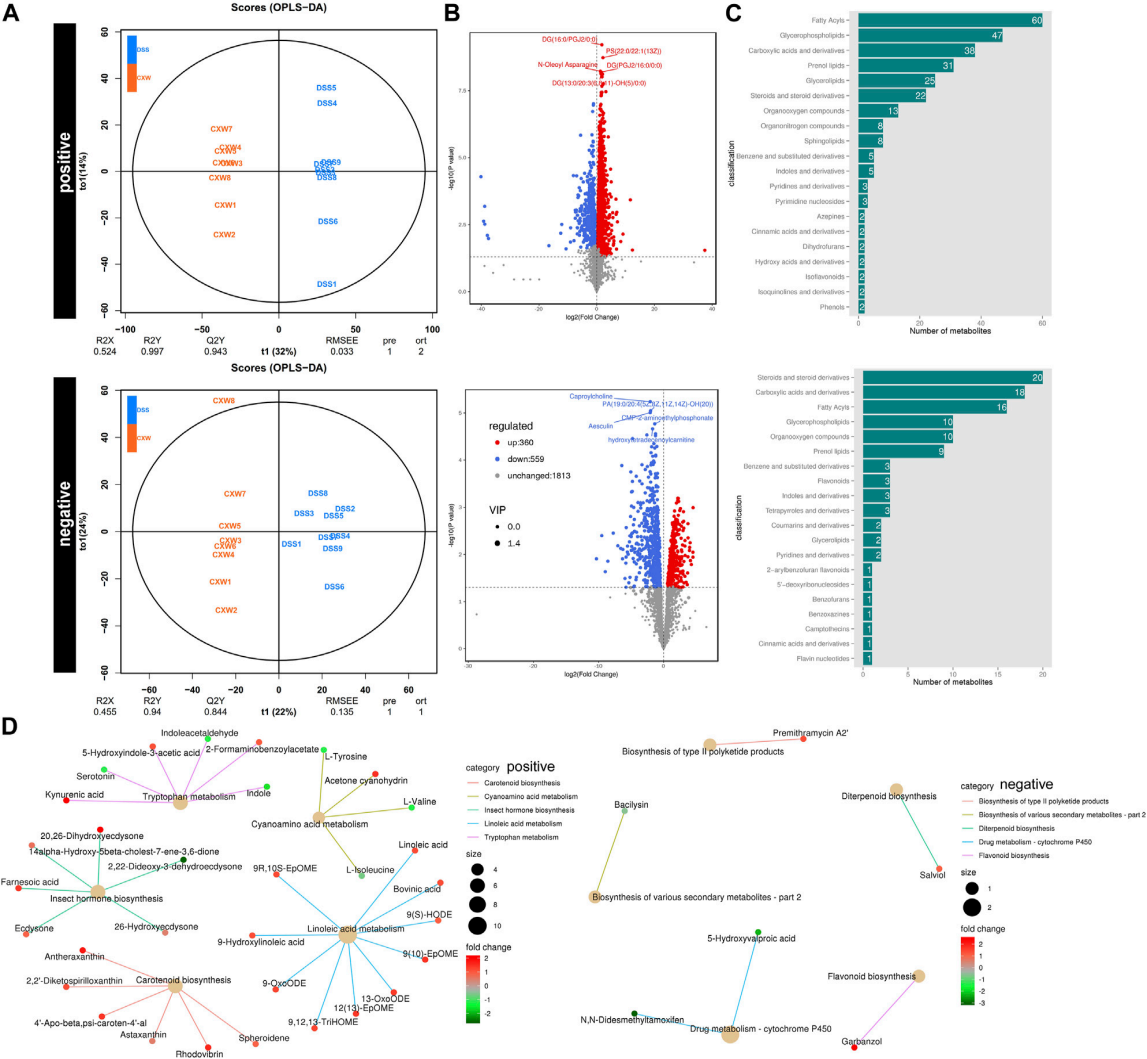
Untargeted metabolomics of UC murine models after CW treatment. OPLS-DAOPLA-DA **(A)**, volcano chart **(B)**, HMDB enrichment **(C)**, and KEGG pathways **(D)** present the clusters of gut microorganisms between UC and CW-treated colitis mice under positive and negative modes.

OPLS-DAOPLA-DA combined with a permutation test also validated the differential metabolites between the DSS and DSS-CW (1.44 g/kg) groups with Q2Y (0.943, 0.844) and R2Y (0.997, 0.94) (Figure 6A). The top five regulated metabolites are listed in Figure 6B (Supplementary Table S5). The differential metabolites between the CW and DSS groups were associated with carboxylic acids and derivatives, glycerophospholipids, fatty acyls, and steroids and derivatives (Figure 6C). The administration of the CW abolished the DSS-induced cytochrome P450 activity and DSS-impaired linoleic acid metabolism, and inhibited serotonin levels in mice subjected to colitis (Figure 6D).

### Cao-Xiang-Wei-Kang promoted the alternative activation of peritoneal macrophages

**FIGURE 7:**
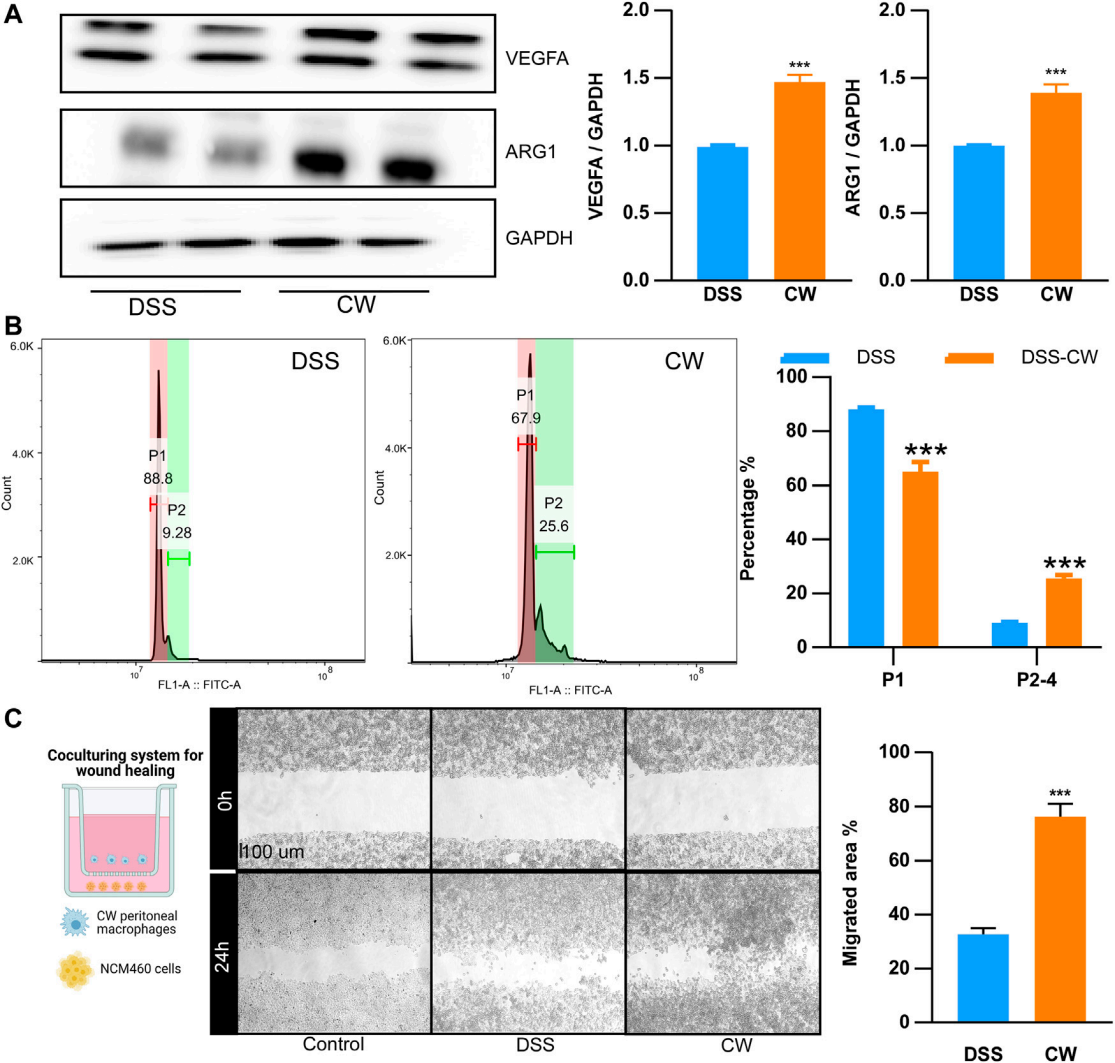
CW facilitates the alternative activation of macrophages. The protein abundance of Arg1 and Vegfa **(A)** in peritoneal macrophages from UC murine models after CW treatment (*n* = 5); phagocytic capacity of peritoneal macrophages from UC murine models after CW treatment **(B)** (*n* = 5); wound healing assay **(C)** showing the migration of NCM460 cells in the presence of peritoneal macrophages from the CW group (*n* = 5). *indicates a statistical difference from the DSS group.

Peritoneal Mφs were collected from UC murine models. The CW formula increased the protein abundance of Vegfa and Arg1 (Figure 7A). As shown in microparticle experiments, the CW treatment increased the proportion of P2–P4 from 9.28% to 25.6% (Figure 7B), suggesting an enhanced M2 transition and alleviated inflammatory reactions. Moreover, NCM460 cells were co-cultured with the CW peritoneal Mφs for 24 h. The wound healing experiment showed an increased migration in the CW group, confirming the favored wound healing capacity of M2 Mφs (Figure 7C).

### Cao-Xiang-Wei-Kang formula rescued the death of intestinal stem cells

**FIGURE 8:**
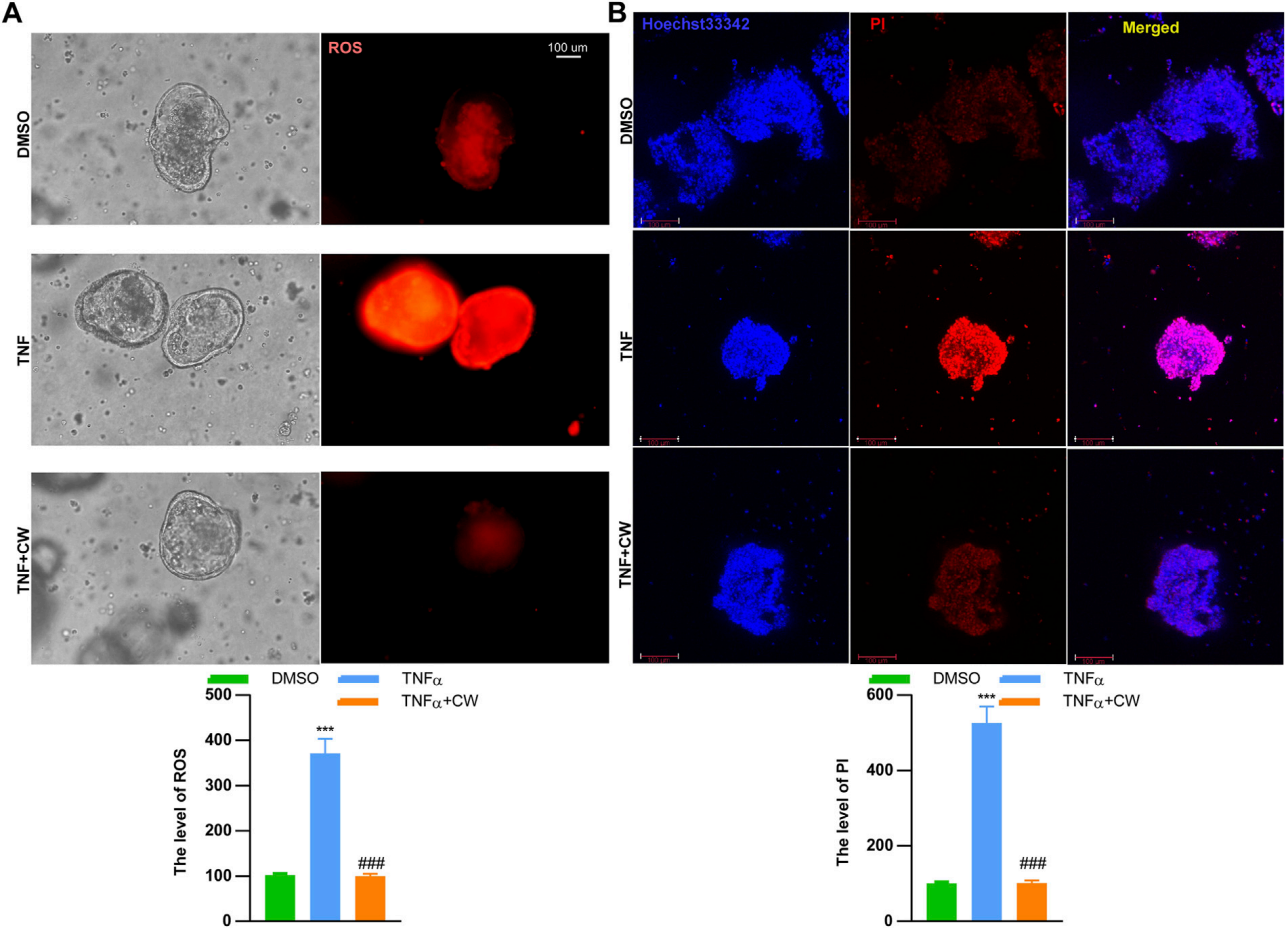
CW serum rescues inflammation-induced cell death of intestinal organoids. The mitochondrial stress **(A)** in TNFα-treated intestinal organoids with the CW serum for 24 h; Hoechst 33342/PI staining **(B)** (*n* = 5). *indicates a statistical difference from the DMSO group; ^#^indicates a difference from the TNFα group.

IOs are composed of nearly all the intestinal cell types, including epithelial cells, endocrine cells, and paneth cells, hence being considered an ideal tool to reflect the status of epithelial recovery. We treated IOs with TNFα (20 ng/ml, 24 h) to construct an *ex vivo* UC cellular model. The serum was isolated from murine colitis models subjected to the CW treatment and healthy blank controls, and both doses of CW serum did not induce apoptosis and impaired the viability of NCM460 cells (Supplementary Figure S3). TNFα treatment increased the ROS level, and this elevation was diminished by the CW serum (Figure 8A). Moreover, TNFα-induced IO death was also rescued after the administration of the CW serum (Figure 8B).

## Discussion

UC occurs mainly in the colon due to a broad spectrum of factors including the microbiome, genetic susceptibility, diet, host immune status, and environment. Patients with UC suffer from mental disorders and physical disadvantages, for example, rectal bleeding, abdominal pain, malnutrition, and diarrhea, with a higher risk of developing CAC than non-UC cohorts (Weingarden and Vaughn, 2017).

Given the rule of “Jun-Chen-Zuo-Shi” in TCM formula theories (Wang et al., 2020a) and the analysis of network pharmacology, SR was responsible for 35% of the weight and 36.7% of the targets in the CW formula and was, thus, considered the major ingredient “Jun,” the targets of which were enriched in infections, cancers, the IL-17 signaling pathway, and neurotransmitter receptor activity, as well as liver diseases including hepatitis and non-alcoholic fatty liver disease. As a potent anti-inflammatory and anti-oxidant ingredient that can treat DSS-induced colitis (Kim et al., 2011; Hou et al., 2018), SR is also able to restrain the colonization of pathological microbes (Sung et al., 2004) and protects from liver injury (Paudel et al., 2018).

OS, Cb, and GB are natural organs from oyster, sepia, and *Gallus gallus domesticus Brisson*, respectively. Since each occupied about 20% of the weight of the CW formula, they were classified as “Chen.” Both OS and Cb are mainly composed of calcium carbonate that exerts an anti-erosive effect against hydrochloric acid from gastric juice (Scandiffio et al., 2018), as well as of chitin (Scandiffio et al., 2018; Kintsu et al., 2020), which promotes the proliferation and differentiation of stem cells (Ho et al., 2021), thereby facilitating wound healing (Francesko and Tzanov, 2011; Lim et al., 2015; Qiu et al., 2020). Noteworthy is the regulated protein processing by OS and Cb predicted by the KEGG analysis, implying that they work synergistically with GB, in which protein is the predominant component. Additionally, OS also improves the host immunity (Malhotra and Kang, 2013; Shi et al., 2021) by increasing SMAD1/5/8 expression (Ho et al., 2021), while the anti-oxidant Cb (Ramasamy et al., 2014) has been reported to protect against gastric ulcers (Qiu et al., 2020) and functions as a nutritional supplementation due to high-soluble amino acid and mineral levels in Cb (Ramasamy et al., 2014). KEGG analysis demonstrated that OS focused on responses to nutrients and hyperoxia, and gastrointestinal functions, including pancreatic secretion, and Cb contributed to re-epithelization by regulating tissue structure homeostasis, which was conducive to the integrity of the endothelial barrier. Consistent with OS and Cb, GB has a historical application in the treatment of ulcers and inflammatory disorders due to its anti-inflammatory, detoxifying, and anti-oxidant effects (Mahmoudi et al., 2013; Pan et al., 2016; Gao et al., 2020; Xiao et al., 2021), as well as the wound healing–promoting property (Salari et al., 2016; Yoo et al., 2017). The targets of GB had a strong correlation with inflammatory reactions, such as TNF, IL-17, HIF-1 signaling pathways, carcinogenesis, and VEGF-regulated migration, as well as responses to oxidative stress, toxic substance, and chemical stress. Apparently, OS and Cb are preferentially improving gastrointestinal functions and epithelial recovery, and providing nutrients, whereas GB shows a potent anti-inflammatory effect. “Chen” improves and expands the efficacy of the CW formula.

DC and FS were considered “Zuo” since they took only 15% of the whole formula. It is worth noting that DC attenuates the progression of DSS-induced colitis (Xie et al., 2020; Zhou et al., 2020), owing to its potent pharmacological activities, for instance, anti-CAC (Hu et al., 2018), anti-inflammation (Rayan et al., 2011; Butturini et al., 2014), and anti-pathogens (Tang et al., 2010; Hasson et al., 2013). Moreover, gastrointestinal spasms (Guo et al., 2014) and diarrhea (Mu et al., 2017) that occur in patients with IBD and murine colitis models could also be alleviated by DC. The anti-bacterial (Iranshahi et al., 2008) and anti-cancer activities (Zhang et al., 2015; Wang et al., 2020b) of FS added to the capacity for combating dysbiosis and preventing CAC. “Zuo” highlights the therapeutic value of the formula, owing to its potent pharmacological activities, and the low dose would not raise toxicity concerns.

Collectively, we summarized the therapeutic strategy of the CW formula from three aspects. To begin with, SR, GB, and DC have anti-inflammatory and anti-oxidant activities, all of which contribute to the resolution of inflammation and inflammation-compromised epithelial barrier function and pain (Baskol et al., 2008), as evidenced by the markedly decreased IL-1b expression and CASP3-dependent pyroptosis (Naji et al., 2016; Li et al., 2022) in murine colitis models subjected to the CW therapy. Mφs are a paradigm of immune cells with a highly plastic capacity able to change their phenotypes in response to environmental cues. M1 Mφs are responsible for initiating innate immunity, while M2 Mφs favor the resolution of inflammation and wound healing (Remmerie and Scott, 2018; Soto-Heredero et al., 2020). CW administration enhanced the expression of M2 markers, Arg1, and VEGFa, indicating an inhibited inflammatory response and a favored re-epithelization.

Second, bloody stool occurs in patients with UC and exacerbates the pain and mental suffering (Bounds and Kelsey, 2007), which could be rescued by accelerated wound healing accomplished by OS, Cb, and GB. Our *in vitro* experiments corroborated that TNFα exacerbated ROS production and induced necroptosis and pyroptosis, which was ameliorated by the CW serum. Moreover, the CW treatment increased VEGFa expression, which is pivotal for mucosal healing and recovery, and CW-treated Mφs improved NCM460 cell migration. Altogether, the CW formula was able to stop gastrointestinal bleeding and help mucosal recovery.

Third, an efficacious modulation of the gut microbiome was accomplished by SR and DC. The components of SR are against several pathogens including *Clostridium perfringens* (Ali et al., 2021), and the broad spectrum of the anti-bacterial activity of DC indicates the therapeutic value in combating infections (Munyaka et al., 2016). In full accordance with our results, DSS-induced colitis decreases the richness and diversity of the gut microbiota, both of which were reversed by the CW formula, highlighting its contribution to the remodeling of gut microbiota. An imbalanced gut commensal flora often begins with an increased prevalence of Proteobacteria and *Bacteroides thetaiotaomicron,* which have been considered potential diagnostic signatures of dysbiosis, depression, and colitis (Shin et al., 2015; Wang et al., 2022), and the CW therapy considerably suppressed their colonization. Moreover, DSS treatment induced the colonization of the genera *Escherichia* and *Shigella*, which is involved in the development of UC into CAC (Tang et al., 2020), which was also inhibited by CW treatment. Additionally, the increased relative abundance of *Dubosiella* and *Lachonospiraceae NK4A136, Mucispirillum schaedleri*, and *Lachnospiraceae* by CW is negatively correlated with the levels of inflammation-promoting cytokines (Wan et al., 2021) and favors gut barrier integrity (Herp et al., 2019; Ma et al., 2020). Altogether, CW treatment restores the colonization of gut commensal flora and plays a protective role in the gut.

Last but not least, the spasmolytic and anti-diarrheal effect of DC, the immunity-orchestrated OS, the nutrition-supplemented Cb, and the hepatoprotective role of DC (Ge et al., 2020) and SR (Paudel et al., 2018) underlie the success of the CW formula in colitis intervention. Moreover, since most TCM formulas have multiple herbs functioning in a synergistic or counteracting mode to achieve homeostasis, drug resistance and metabolism are the major challenges for drug efficacy and safety. Among the six ingredients, Cb, GB, and OS are atoxic. The dosage of DC in the CW formula for adults was 0.3 g/d, which is far less than the recommended level 3–6 g/d in Chinese Pharmacopoeia 2020 (Huang et al., 2021) and hence resolves the concern regarding toxicity (Bei-Xuan et al., 2016). The potential hepatoxicity of SR (Yang et al., 2021) and FS might be counteracted by the detoxifying effect of GB (Mahmoudi et al., 2013; Pan et al., 2016; Gao et al., 2020; Xiao et al., 2021). As a result, 16S rRNA gene sequencing results confirmed that the CW therapy inhibited the prevalence of *Shigella sonne*, which contributes to drug resistance (Starling, 2017). As seen in our metabolomic analysis, Cytochromes P450 (CYPs), enzymes with catalytic activities regulating drug metabolism and toxicity (Guengerich et al., 2016), were enhanced during DSS-induced colitis, which was reversed after the CW treatment, suggesting a resolved issue regarding toxicity.

Collectively, the anti-colitis effect of the CW formula is attributed to multiple active components. We constructed a target–component network utilizing hub genes calculated by network pharmacology and their corresponding components and found that all of the six ingredients, which are 29 components, were associated with the hub genes, which might be the key active components of the CW formula to treat colitis and needs further pharmacokinetic investigation.

## Conclusion

We posit that the CW formula is an efficient formula to treat colitis by combining low-toxic ingredients with atoxic medicinal animal organs, and it suppresses inflammation and the prevalence of pathogens, with the concomitant improvements in gut immunity and nutrient supply. Nevertheless, the therapeutic effects of each component merit closer investigation, which could pave the way for adding to the understanding of this formula.

## Data Availability

The datasets presented in this study can be found in online repositories. The names of the repository/repositories and accession number(s) can be found at: https://www.ncbi.nlm.nih.gov; PRJNA827781.

## Supplementary material

SUPPLEMENTARY TABLE S1 LC-MS/MS identified active components of CW.

SUPPLEMENTARY TABLE S2 Targets of CW.

SUPPLEMENTARY TABLE S3 Alpha diversity indices of the gut microbiota after CW treatment.

SUPPLEMENTARY TABLE S4 Altered metabolites in the fecal samples from colitis mice.

SUPPLEMENTARY TABLE S5 Altered metabolites in the fecal samples from colitis mice after CW treatment.

SUPPLEMENTARY FIGURE S1 LC-MS/MS identified CW components.

SUPPLEMENTARY FIGURE S2 Alteration in the microbiome profile in murine colitis models OPLS-DAOPLA-DA ,**(A)** permutation test **(B)**, and volcano chart **(C)** showing the metabolite clusters between saline and colitis mice under positive and negative modes; KEGG pathways **(D)**.

SUPPLEMENTARY FIGURE S3 MTT assay evaluating the toxicity of the CW serum PI staining **(A)** and MTT assay **(B)** of the NCM460 cells with different concentrations of the CW serum (CW-low: 0.7 g/kg ; high: 1.4 g/kg).

## Abbreviations

IBD, inflammatory bowel disease; CAC, colitis-associated carcinogenesis; CW, Cao-Xiang-Wei-Kang; TCM, traditional Chinese medicine; PPIs, protein–protein interactions; UC, ulcerative colitis; Mφs, macrophages; ISCs, intestinal stem cells; IOs, intestinal organoids; KM, Kaplan–Meier; CC, closeness centrality; BC, betweenness centrality; DC, degree centrality; EC, eigenvector centrality; LAC, local average connectivity; NC, network centrality; GO, Gene Ontology; KEGG, Kyoto Encyclopedia of Genes and Genomes; TNFα, tumor necrosis factor-α; ROS, reactive oxygen species; Casp3, caspase 3; IL-17, interleukin-17; IL-1b, interleukin-1b.
