# Supplementary figures and images for: The Cao-Xiang-Wei-Kang formula attenuates the progression of experimental colitis by restoring the homeostasis of the microbiome and suppressing inflammation
Source: Front Pharmacol. 2022 Sep 20;13:946065. doi: 10.3389/fphar.2022.946065 (PMC9530714; doi:10.3389/fphar.2022.946065)

## Slide 1
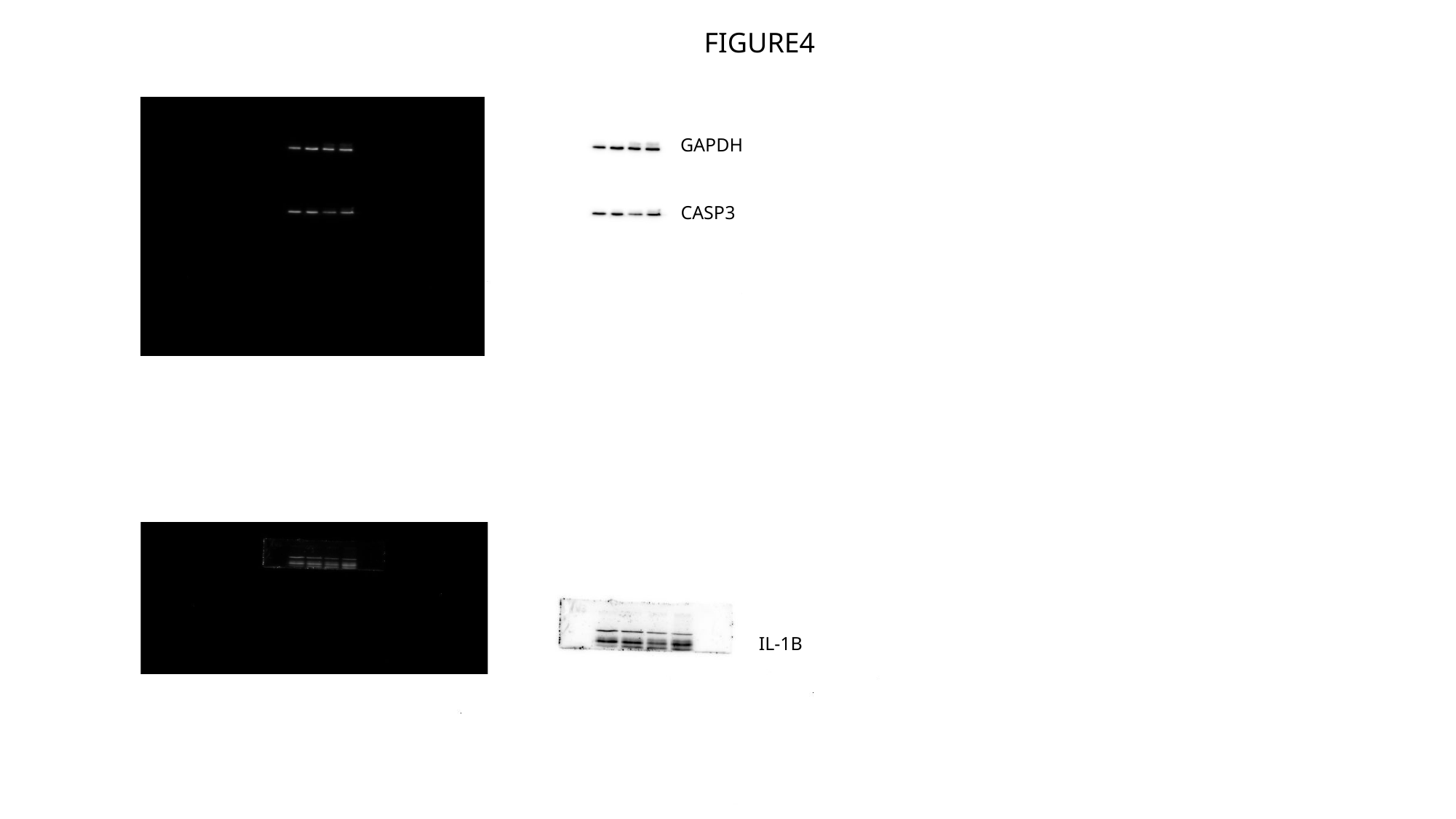

FIGURE4
GAPDH
CASP3
IL-1B

## Slide 2
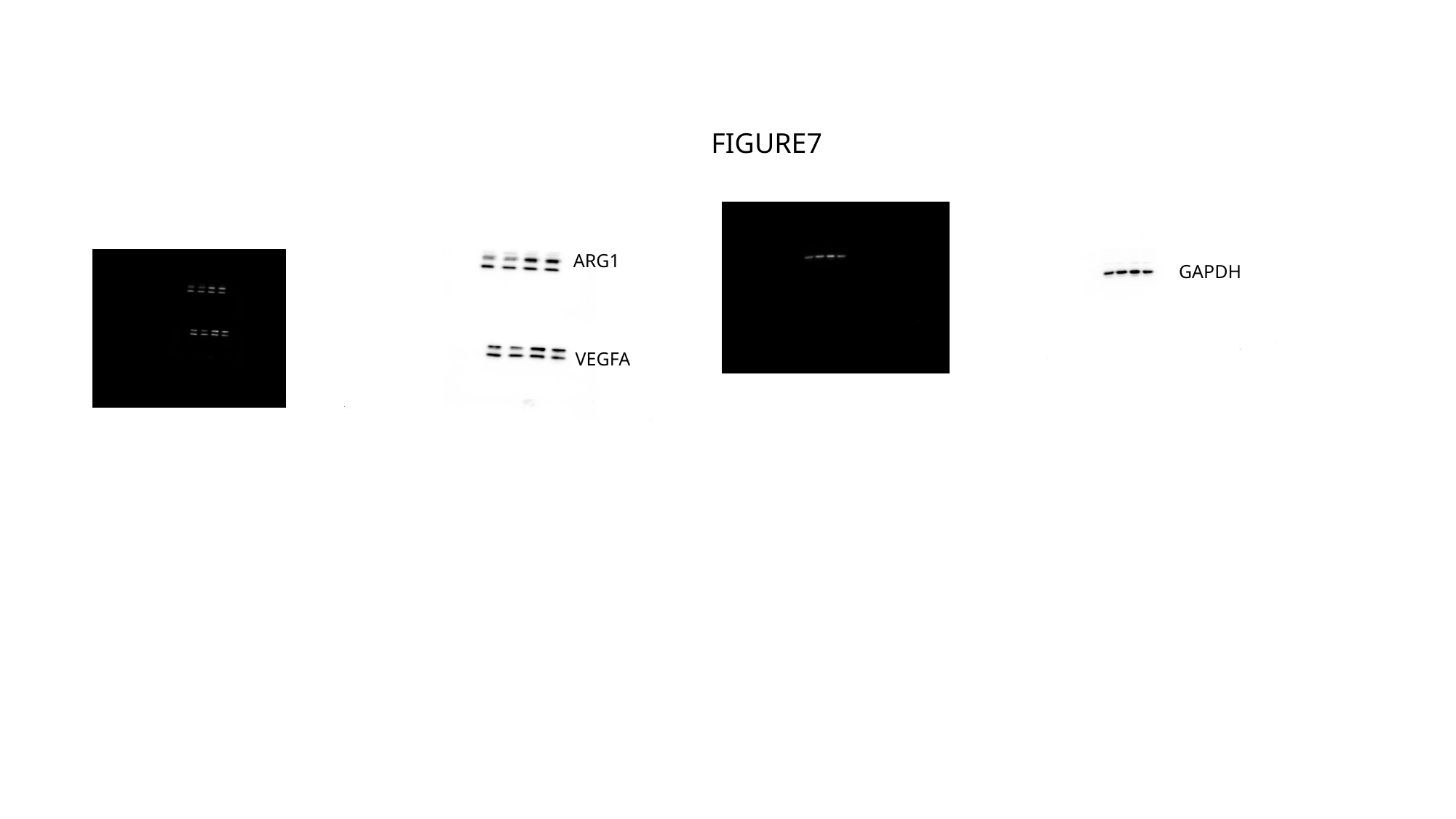

FIGURE7
ARG1
GAPDH
VEGFA

Supplement: Supplementary file 3 [file Presentation1.PPTX]

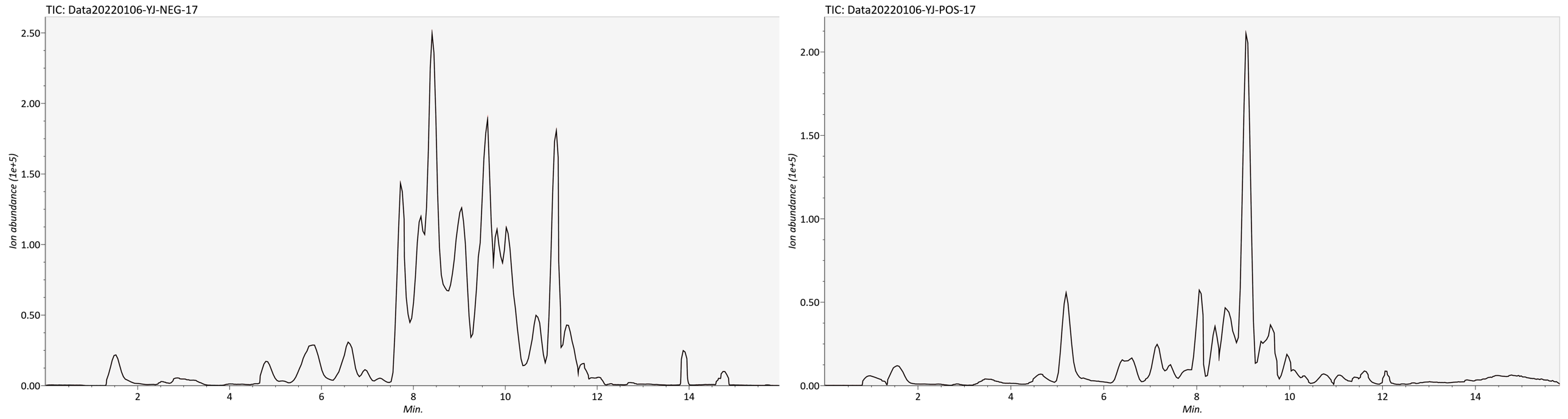

Supplement: Supplementary file 4 [file Image1.JPEG]

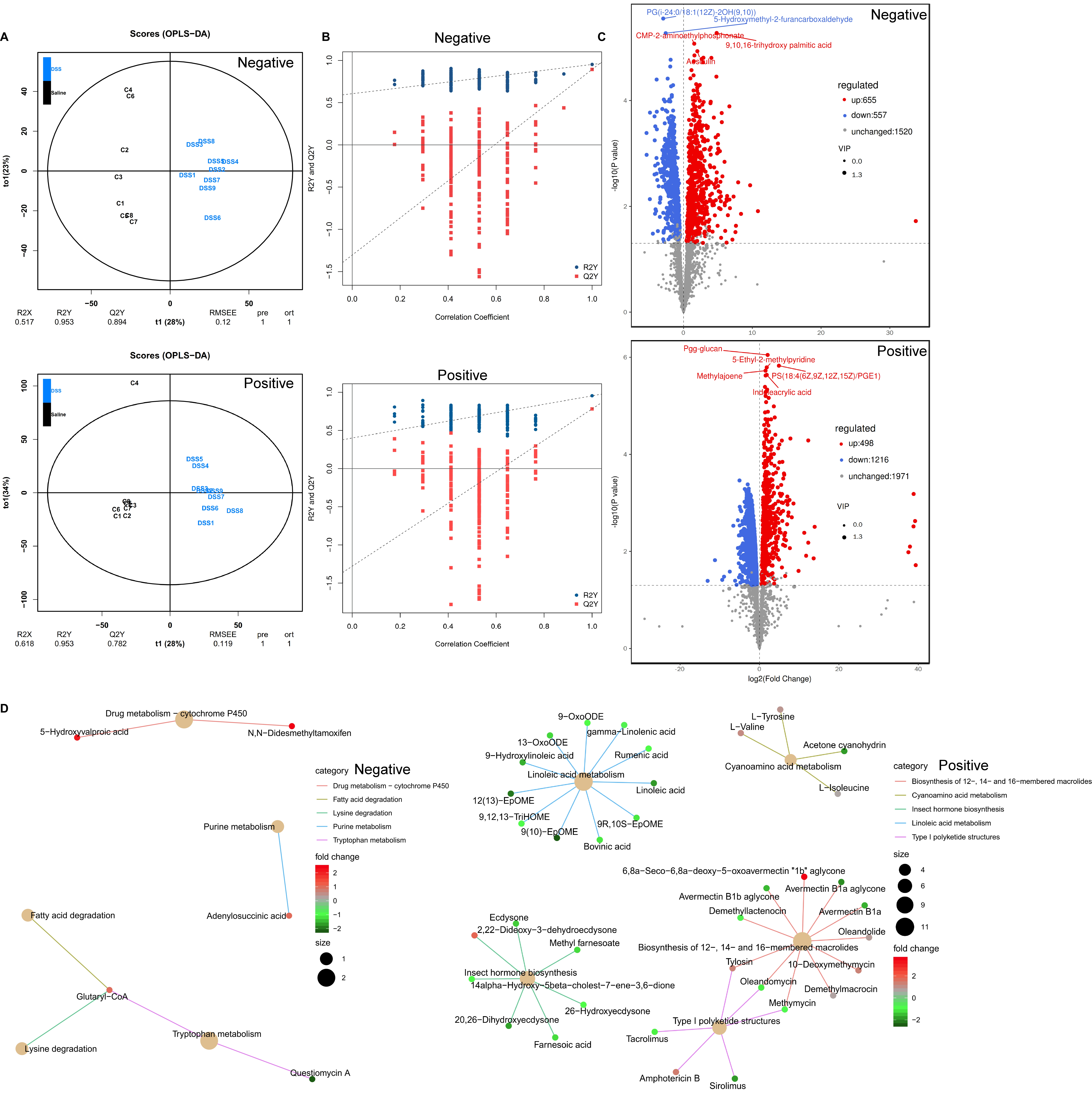

Supplement: Supplementary file 5 [file Image2.JPEG]
